# Tissue-Derived Small Extracellular Vesicles: Emerging Regulators of Inter-Organ Crosstalk in Health and Disease

**DOI:** 10.3390/metabo16030148

**Published:** 2026-02-24

**Authors:** Yin-Qiong Huang, Chuan-Yu Zhong, George Burley, Yan-Chuan Shi, Shu Lin

**Affiliations:** 1 Department of Endocrinology, The Second Affiliated Hospital of Fujian Medical University, Quanzhou 362002, China; yinqiongh@fjmu.edu.cn (Y.-Q.H.); zhongcy666666@163.com (C.-Y.Z.); 2 Neuroendocrinology Group, Garvan Institute of Medical Research, Sydney, NSW 2010, Australia; g.burley@student.unsw.edu.au; 3 Brain & Body Metabolic Lab, Victor Chang Cardiac Research Institute, Lowy Packer Building, 405 Liverpool Street, Darlinghurst, NSW 2010, Australia; 4 Centre of Neurological and Metabolic Research, The Second Affiliated Hospital of Fujian Medical University, Quanzhou 362002, China

**Keywords:** small extracellular vesicles (sEVs), extracellular vesicles (EVs), inter-organ crosstalk, biomarker, therapeutic delivery

## Abstract

Small extracellular vesicles (sEVs; commonly referred to as “exosomes” in many studies) are nanoscopic messengers released by healthy and diseased cells that mediate intercellular communication by transferring proteins, lipids, and nucleic acids to local or distant recipient cells. In this narrative review, we synthesize recent evidence linking tissue-derived sEVs to neurological disorders (including neurodegeneration and traumatic brain injury), metabolic syndrome, cardiovascular diseases, cancers, and bone diseases, with a particular emphasis on CNS–periphery crosstalk across the blood–brain barrier. Compared with prior reviews that focus on single organ systems, we highlight cross-disease, cross-tissue mechanisms and summarize candidate biomarker cargos and therapeutic strategies in dedicated tables. While accumulating data support brain–body communication via sEVs, the concept of CNS-derived sEVs acting as a “third central efferent pathway” is presented here as an emerging hypothesis that complements—rather than replaces—neuronal and endocrine signaling. Overall, tissue-derived sEVs represent a promising but still evolving platform for diagnostic and therapeutic innovation, warranting standardized isolation/characterization and further clinical validation.

## 1. Introduction

Extracellular vesicles (EVs) are lipid-bilayer nanoparticles released by cells, spanning multiple size ranges and biogenesis routes. In this review, we primarily discuss small EVs (sEVs; commonly 30–150 nm), a category that includes exosomes, but we use the term “exosome” only when endosomal origin is experimentally supported. This terminology follows the Minimal Information for Studies of Extracellular Vesicles (MISEV2023) recommendations, which emphasize rigorous operational definitions based on isolation and characterization rather than assumed biogenesis [1].

sEVs can be classified by source (e.g., cultured cells, specific tissues/organs, or circulating biofluids). Tissue-derived sEVs reflect in vivo states and carry proteins, lipids, and nucleic acids (DNA, mRNA, microRNA, lncRNA), thereby acting as ‘mini snapshots’ of their parent cells that can modulate recipient-cell metabolism, immunity, and stress responses locally or systemically [2,3]. sEVs are readily detected in plasma/serum and diverse biofluids (urine, saliva, synovial fluid, amniotic and pleural fluids, breast milk), enabling tissue-informed liquid biopsy approaches. Because of their diverse origins and targets, sEVs have been implicated across neurodegenerative diseases, traumatic brain injury (TBI), metabolic disorders, cardiovascular diseases, cancers, and bone diseases [3,4,5,6,7]. However, terminology, isolation methods, and mechanistic interpretations vary substantially across the literature, which can complicate cross-study comparison and clinical translation.

Diseases/conditions discussed in this review were selected based on (i) high clinical burden and prevalence, (ii) evidence that tissue-derived sEV cargos are measurable in accessible biofluids, and (iii) representative mechanistic paradigms of inter-organ communication (CNS–periphery, metabolic–immune, vascular remodeling, tumor microenvironment, and joint/bone remodeling).

Here, we provide a narrative synthesis of recent advances on how tissue-derived sEVs contribute to disease onset and progression through intercellular communication and remote regulation of target organs. We summarize (i) candidate sEV cargos linked to diagnosis/prognosis, and (ii) sEV-based or sEV-informed therapeutic strategies, while explicitly distinguishing experimentally supported evidence from emerging hypotheses.

## 2. Literature Search Strategy and Study Selection

This narrative review is supported by a structured literature search to ensure methodological transparency. Relevant studies on EVs/sEVs in inter-organ communication and clinical diseases were identified using PubMed, Web of Science, and Scopus (last search: 5 February 2026) with keywords including “extracellular vesicle”, “small extracellular vesicle”, “exosome”, “biomarker”, “therapy”, and disease-specific terms.

We prioritized English-language original studies and high-quality reviews with rigorous EV characterization (particle sizing, EV markers, contamination controls) and, when available, in vivo validation. Studies were not considered when EV identity was insufficiently characterized or conclusions relied mainly on non-vesicular fractions.

Representative sEV/EV biomarker cargos linked to diagnosis and prognosis across major diseases are summarized in Table 1.

## 3. Exosomes and Neurodegenerative Diseases

### 3.1. The Impact of Periphery-Derived sEVs on the Central Nervous System (CNS)

**Figure 1:**
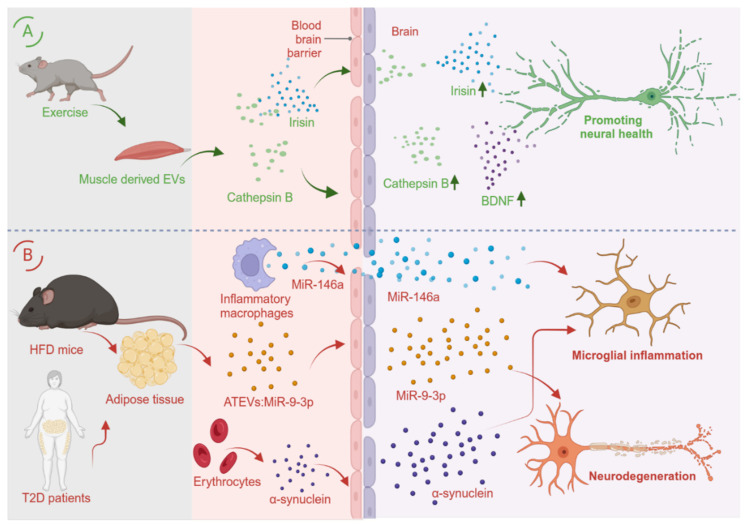
Impact of peripheral-derived sEVs/EVs on the CNS. (**A**): After exercise, muscle-derived EVs can carry myokines (e.g., irisin, cathepsin (**B**)) across the BBB and into the hippocampus, supporting neurogenesis and cognitive function. (**B**): In metabolic and neurodegenerative contexts, ATEVs (e.g., miR-9-3p-enriched) can accumulate in hippocampal neurons and impair synaptic integrity; inflammatory macrophage-derived EVs carrying miR-146a can activate microglia and reduce Aβ clearance in AD; and erythrocyte-derived EVs enriched in alpha-synuclein can cross the BBB and promote microglial inflammation in PD. Created with BioRender.com.

Exercise promotes metabolic and cognitive benefits partly through inter-organ communication that reaches the CNS. Accumulating evidence suggests that circulating EVs/sEVs released from peripheral tissues can reach the brain and modulate neuronal and glial function. Candidate mediators include metabolites and cytokines/myokines that support synaptic plasticity, neuroprotection, and cognition [20,21,22,23,24]. Brain-derived neurotrophic factor (BDNF) is strongly linked to exercise-associated hippocampal plasticity, and endurance exercise induces hippocampal BDNF via the PGC-1alpha/FNDC5 (irisin) pathway [25]. Because free peptides and nucleic acids may be unstable in the circulation, sEVs provide a protective carrier that can facilitate blood–brain barrier (BBB) transit and targeted delivery [26]. Consistent with this, exercise increases circulating sEVs that carry muscle-associated cargos, and cathepsin B has been implicated as an exercise-induced factor that contributes to enhanced hippocampal neurogenesis and memory [27,28]. Moreover, cathepsin B can promote amyloid-beta (Aβ) clearance pathways and has been linked to neuroprotective effects in Alzheimer’s disease models (Figure 1A) [29,30].

Peripheral-derived sEVs can also exert detrimental effects on the CNS. In Alzheimer’s disease (AD), inflammatory macrophage-derived sEVs carrying miR-146a can drive microglial polarization toward a pro-inflammatory state and reduce Aβ clearance through TLR-related tolerance mechanisms, potentially linking peripheral inflammation (e.g., diabetes, aging, cardiovascular disease) to elevated AD risk [31]. In Parkinson’s disease (PD), erythrocyte-derived EVs enriched in alpha-synuclein can cross the BBB—particularly under systemic inflammatory conditions—and amplify microglial activation, providing a plausible route for peripheral contributions to synucleinopathy progression [11].

Emerging work also implicates adipose tissue-derived EVs in brain dysfunction during metabolic disease. In obese mice and in patients with type 2 diabetes, adipose tissue-derived EVs (ATEVs) can access the hippocampus and promote synaptic injury and cognitive impairment; miR-9-3p has been identified as a key cargo contributing to these phenotypes, and its inhibition mitigates ATEV-associated neurotoxicity [32]. Together, these findings position peripheral sEVs as both mechanistic mediators and candidate biomarker/therapeutic targets in neurodegenerative disorders (Figure 1B).

### 3.2. CNS-Derived sEVs in Neuronal Health and Neurodegeneration

CNS-derived sEVs are produced by neurons, oligodendrocytes, astrocytes, and microglia and contribute to homeostatic support as well as disease propagation. Oligodendrocyte-derived sEVs can deliver metabolic enzymes, myelin-associated proteins, and regulatory RNAs that support axonal transport and long-term neuronal maintenance in vivo [33]. Astrocyte- and neuron-derived sEVs have also been reported to modulate synaptic activity and stress resilience through transfer of trophic factors and microRNAs, whereas microglial sEVs can shape inflammatory tone and phagocytic programs in recipient cells [34].

In neurodegenerative diseases, pathological sEV signaling can facilitate the intercellular transfer of neurotoxic proteins (including Aβ, tau, and alpha-synuclein), thereby amplifying protein aggregation, neuroinflammation, and progressive neuronal loss [8,9,10]. Accordingly, CNS-derived sEV cargos are being explored as liquid-biopsy candidates that may better reflect cell-type-specific pathology than bulk plasma markers.

### 3.3. CNS-Derived sEVs and Peripheral Tissues: An Emerging (Putative) Third Efferent Route

Compared with the effects of peripheral EVs/sEVs on the brain, the impact of CNS-derived sEVs on peripheral organ function remains less explored, and direct causal proof is still limited. Nevertheless, emerging studies support BBB transit of CNS-derived EVs and their association with peripheral phenotypes.

One in vivo example links CNS-derived EVs to distal bone remodeling in Alzheimer’s disease. Brain-derived EVs have been detected in peripheral bone after systemic circulation, and EVs isolated from AD mouse brains were reported to shift bone marrow stromal cell differentiation from osteogenesis toward adipogenesis, contributing to bone–fat imbalance. Mechanistically, miR-483-5p was enriched in AD brain EVs and appeared to inhibit Igf2 signaling in recipient cells [35]. While these findings support feasibility of CNS-to-bone EV transfer, additional studies are needed to define biodistribution, dosing, and causality.

**Figure 2:**
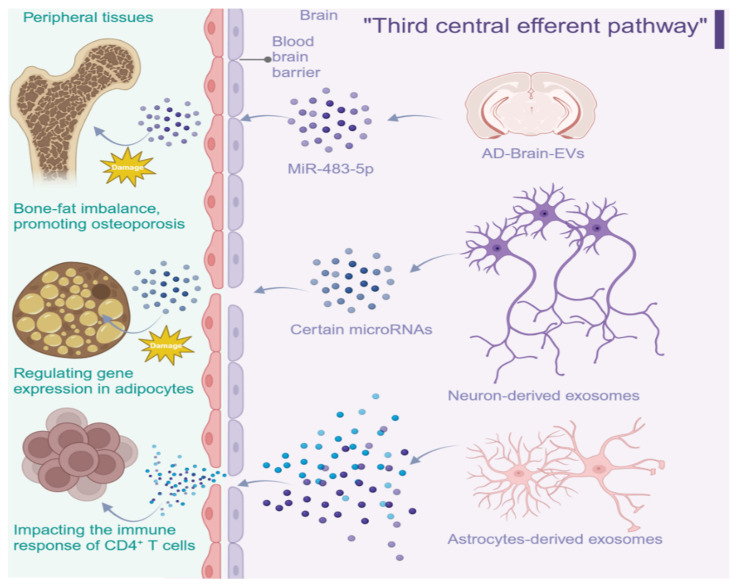
CNS-derived sEVs can cross the BBB and reach peripheral targets (e.g., bone, adipose tissue, immune cells), supporting an emerging hypothesis of a putative “third central efferent route” that complements classical neuronal and endocrine pathways. Created with BioRender.com.

Beyond bone, neuronal EV-associated microRNAs have been associated with transcriptional regulation in peripheral metabolic tissues such as adipocytes, potentially influencing lipid metabolism and insulin sensitivity [36]. CNS-derived EVs may also modulate peripheral immunity: astrocyte-derived EVs with distinct activation states can differentially shape CD4^+^ T-cell responses in vitro and in multiple sclerosis cohorts [37]. Collectively, these observations support an emerging hypothesis that CNS-derived sEVs may act as a putative “third central efferent route” that complements neuronal and endocrine signaling (Figure 2), but key gaps remain regarding targeting specificity and physiological relevance.

## 4. sEVs and Traumatic Brain Injury (TBI)

### 4.1. sEV-Based Therapeutic Strategies in TBI

Clinically, blood-based protein biomarkers such as glial fibrillary acidic protein (GFAP) and ubiquitin C-terminal hydrolase L1 (UCH-L1) have been cleared by the U.S. Food and Drug Administration (FDA) to assist in the evaluation of adults with suspected mild traumatic brain injury (TBI), particularly in guiding decisions regarding the need for head CT imaging [38]. Although these biomarkers reflect astroglial and neuronal injury, respectively, they primarily indicate generalized structural brain damage and provide limited insight into the specific cellular sources and intercellular signaling mechanisms that drive post-traumatic pathology. In this context, small extracellular vesicle (sEV)–associated cargos may offer complementary and more cell-specific information by carrying molecular signatures derived from neurons, glia, and endothelial cells, thereby reflecting active intercellular communication processes in TBI [39].

CNS-derived sEVs have been shown to modulate post-TBI inflammation and repair by transferring regulatory microRNAs and trophic factors to microglia, neurons, and endothelial cells. Long et al. reported that activated astrocyte-derived sEVs enriched in miR-873a-5p attenuated microglia-mediated neuroinflammation and improved neurological outcomes after TBI, at least partly through inhibition of NF-κB signaling [40]. Similarly, microglia-derived sEVs enriched in miR-124-3p reduced neuroinflammation and supported neuronal survival in repetitive mild TBI models [41,42].

Beyond CNS sources, stem cell-derived sEVs are increasingly investigated as cell-free therapeutic candidates. In rat TBI models, mesenchymal stem cell (MSC)-derived sEVs (commonly from bone marrow-derived MSCs in preclinical studies) improved sensorimotor and cognitive function, reduced hippocampal neuronal loss, and promoted angiogenesis and neurogenesis, with a therapeutic window of at least 7 days post-injury [43]. Neural stem/progenitor cell-derived sEVs can deliver pro-angiogenic factors (e.g., VEGF) and regulatory RNAs, promoting vascular repair and reducing neuronal apoptosis, further supporting CNS regeneration in TBI [44].

Collectively, these studies identify mechanistically relevant sEV cargos (miR-873a-5p, miR-124-3p, VEGF-related programs) that may inform future engineered or targeted sEV-based interventions.

### 4.2. Potential Pathogenic Mechanisms and Neuroinflammation Mediated by sEVs

In addition to protective roles, sEVs may contribute to secondary injury by amplifying neuroinflammatory circuits and propagating pathological protein signaling. After TBI, increased miR-21-5p has been reported in neuronal and microglial compartments. Neuron-derived sEVs enriched in miR-21-5p can be internalized by microglia, promoting a pro-inflammatory phenotype, increasing cytokine release, and exacerbating neuronal stress and tau phosphorylation in experimental systems [12].

Beyond microRNA signaling, sEV-associated tau species are emerging as potential mediators and biomarkers of TBI-related neurodegeneration. Brain-derived sEVs carrying phosphorylated tau have been detected following experimental and clinical TBI and may contribute to the spread of tau pathology and long-term cognitive decline [13]. Furthermore, aging alters brain-derived sEV cargo and inflammatory tone. sEVs from aged animals have been reported to worsen neuroinflammatory outcomes after injury compared with sEVs from young donors, suggesting age-dependent differences in sEV-mediated secondary injury mechanisms [45].

## 5. sEVs/EVs in Metabolic Syndrome

Metabolic syndrome is a complex disorder characterized by multiple metabolic abnormalities, including obesity, hypertension, hyperglycemia, and dyslipidemia. Its pathogenesis is intricate, involving a dynamic network of organ crosstalk, in which exosomes play an increasingly recognized role. More broadly, the secreted factors at play in this pathology include hepatokines, myokines and adipokines, the signaling molecules released by liver [46], skeletal muscle [47] and adipose tissue [14], respectively. They play crucial roles in regulating metabolism, inflammation, and various physiological and pathological processes.

### 5.1. Exosomes from Liver and Skeletal Muscle

Liver-derived EVs play a crucial role in glucose regulation. Miotto et al. employed mass spectrometry proteomics to characterize the changes in protein composition of liver-derived EVs from healthy vs diseased livers, namely the transition of healthy liver to non-alcoholic fatty liver disease (NAFLD) and non-alcoholic steatohepatitis (NASH) in mice. Interestingly, liver-derived EVs from both mice and humans improved glycaemic control regardless of underlying liver pathology. This was achieved through increasing insulin secretion and enhancing glucose effectiveness, independent of changes in insulin sensitivity. The cargo of the EVs alone was found to be insufficient to produce these effects, instead being dependent on the presence of transmembrane proteins in the EVs to mediate the interaction with their target cells. The secretion of liver EVs was increased when exposed to high versus low glucose levels, indicating a dynamic role of these EVs in glucose control. Further, this dynamic secretion was suppressed in NAFLD mice. This indicates that liver-derived EVs have an important physiological role in rapidly regulating glycaemic control in response to fluctuating glucose levels [46].

A mouse study has indicated that muscle-derived exosomes may play a crucial role in various aspects of metabolic syndrome. Jalabert et al. found that skeletal muscle-derived exosomes injected into mice could specifically target miR pancreatic islet cells and affect gene expression and proliferation of β cells [48]. Similarly, muscle and liver-derived exosomes from mice with HFD-induced insulin resistance can integrate into the pancreas and modulate gene expressions in islets, causing compensatory β-cell proliferation [49]. This process further highlights the potential of exosomes in mediating interactions between multiple organs and their role in regulating systemic metabolism.

### 5.2. Exosomes from Adipose Tissue

Adipose tissue-derived EVs/sEVs can modulate macrophage polarization and systemic insulin sensitivity, highlighting their role in metabolic dysregulation [50]. Because they often transfer microRNAs, adipose-derived sEVs have been proposed as ‘vesicular adipokines’ that relay metabolic state to distant organs [51]. Importantly, obese phenotypes show distinct sEV microRNA signatures compared with lean controls. For example, obesity and insulin resistance have been associated with enrichment of pro-inflammatory or insulin-desensitizing microRNAs (e.g., miR-155, miR-34a, miR-27a) and depletion of vasculo-protective microRNAs (e.g., miR-126) in circulating or adipose-derived EV fractions, consistent with impaired angiogenesis and heightened inflammation [14,15]. In type 2 diabetes, altered EV abundance and microRNA signaling have also been linked to vascular complications and impaired repair programs [52,53]. Together, these findings support a feed-forward loop in which adipose dysfunction reshapes EV cargo, which in turn exacerbates metabolic pathology [54].

## 6. sEVs/EVs in Cardiovascular Diseases (CVD)

Atherosclerosis, heart failure and acute myocardial infarction (AMI) are common forms of CVD. In recent years, exosomes have been identified as important mediators of cardiovascular health, influencing processes such as angiogenesis, inflammation, and apoptosis [55]. For example, cardiac-derived exosomes can carry cardioprotective factors, promoting cell survival and tissue repair following ischemic injury [56]. Almost all types of cardiovascular cells have been shown to release exosomes and respond to their signals. This includes cardiomyocytes and fibroblasts in the heart, as well as endothelial cells and macrophages in peripheral tissues [57].

### 6.1. Atherosclerosis-Related Exosomes

sEVs/EVs play an important role in the progression of atherosclerosis associated with obstructive sleep apnea–hypopnea syndrome (OSA) [16]. OSA is characterized by recurrent intermittent hypoxia, which promotes oxidative stress and systemic inflammation and is strongly linked to adverse cardiovascular outcomes. Intermittent hypoxia can elevate circulating LDL-C and is reported to shift circulating EV cargo toward a more pro-atherogenic profile (e.g., increased inflammatory signaling, endothelial activation cues, and impaired reparative capacity), thereby promoting endothelial dysfunction and plaque progression [16]. In addition, EVs released from endothelial cells, macrophages, and vascular smooth muscle cells can promote plaque formation by facilitating leukocyte recruitment, enhancing platelet–leukocyte adhesion, stimulating VSMC proliferation/migration, and accelerating apoptosis [58].

### 6.2. Heart Failure-Related Exosomes

With a growing burden of patients suffering from heart failure, there is much interest in discovering new biomarkers for the disease. This is a chronic condition characterized by cardiac hypertrophy and fibrosis, leading to poor heart function and increased risk of complications such as arrhythmias and stroke. A study conducted in rats found that exosomes containing miR-21 (exo-miR-21) are of interest as a possible biomarker in heart failure. Elevated levels of MiR-21 have been observed in failing hearts. While inhibiting or overexpressing miR-21 levels in cardiomyocytes does not appear to influence cardiac hypertrophy, exo-miR-21 derived from fibroblasts may play a critical role in inducing pro-hypertrophic changes in both cardiomyocytes and cardiac fibroblasts [59]. Furthermore, exosomes are implicated in the regulation of cardiac fibrosis, playing an important role in influencing extracellular matrix remodeling [60].

### 6.3. Acute Myocardial Infarction-Related Exosomes

Exosomes have demonstrated significant potential as biomarkers in AMI, offering promise not only for disease diagnosis but also for their potential role in cardiac repair. In vivo research has shown that specific miRNAs, such as miR-204, can be isolated from the exosomes of AMI patients. The expression levels of this miRNAs are closely related to the pathological process of myocardial infarction, suggesting that miRNAs may serve as diagnostic biomarkers [61]. In terms of therapy, stem cell-derived exosomes may be a promising therapy for AMI, enhancing cardiac function, fibrogenesis, and inflammatory responses while reducing cell apoptosis and autophagy. In preclinical animal models, exosome therapy shows potential for these benefits, providing valuable insights for future clinical trials in humans [62].

## 7. sEVs/EVs in Tumors and Neoplastic Diseases

### 7.1. Ovarian and Breast Cancer

sEVs/EVs have emerged as important biomarker candidates and functional mediators in ovarian and breast cancer. In ovarian cancer, tumor-derived EV cargos (e.g., specific miRNAs and proteins) have been proposed for early detection and for monitoring chemoresistance, partly by reprogramming stromal cells and promoting angiogenesis and immune evasion [63]. In breast cancer, EV-associated lncRNAs and miRNAs can promote metastatic niche formation and therapy resistance, while circulating EV cargos are being explored as minimally invasive biomarkers [64].

### 7.2. Lung Cancer

Exosomes have emerged as significant players in lung cancer, serving both as biomarkers for diagnosis and as potential therapeutic vehicles. For instance, Jin et al. used next-generation sequencing technology to validate that exosomal let-7b-5p, let-7e-5p, miR-23a-3p, and miR-486-5p had an area under the curve (AUC) of 0.899 for diagnosing early-stage lung cancer [17]. In addition, the presence of exosomal PD-L1 has been linked to immune evasion in non-small cell lung cancer (NSCLC), emphasizing the role of exosomal components in tumor immunology [63]. Plasma exosomal microRNAs from patients have been identified as potential biomarkers for immunotherapy of NSCLC. A controlled study of patients with advanced EGFR/ALK wild-type NSCLC who received PD-1/PD-L1 inhibitors showed that compared with normal controls, lower levels of exosome-derived hsa-miR-320d, hsa-miR-320c, and hsa-miR-320b may indicate the better efficacy of PD-1/PD-L1 immunotherapy in advanced NSCLCs. In addition, when hsa-miR-125b-5p, a T-cell suppressor in exosomes, is downregulated during immunotherapy, NSCLC patients may gain enhanced T-cell function and respond well [64,65].

### 7.3. Hepatocellular Carcinoma (HCC)

In recent years, an increasing number of studies have shown that EVs play a crucial role in the progression of HCC, with the non-coding RNAs they carry (such as circRNAs and lncRNAs) being particularly important in regulating the tumor microenvironment (TME) [18]. CircRNAs encapsulated within EVs regulate the TME by promoting hypoxic stress response, stimulating angiogenesis, facilitating metabolic reprogramming, inducing inflammatory changes within HCC cells, and triggering tumor immunosuppression. EVs derived from HCC cells transport circRNAs to immune cells, interfering with the activation of immune cells, promoting over-expression of immune checkpoints, thereby regulating the immune response, giving tumor cells immunosuppressive characteristics, and accelerating tumor progression. circRNAs and lncRNAs carried by HCC EVs can affect the proliferation, invasion, metastasis and induction of chemoresistance in HCC cells [66,67]. Tumour cells contain various factors to mediate their release of exosomes. For example, in vitro studies revealed that lncRNA HOTAIR, upregulated in many cancer cells including HCC, promotes exosome secretion in HCC cells by regulating RAB35 and SNAP23 [68]. In addition, Du et al. found that M6 A-mediated up-regulation of circMDK was upregulated in HCC, promoting tumorigenesis and was associated with poor survival in HCC patients. As such, circMDK could act as a biomarker for HCC tumours, and possibly a therapeutic nanotarget [69].

## 8. sEVs/EVs in Bone Diseases

Bone diseases, particularly osteoporosis and osteoarthritis (OA), have also been linked to exosomal contents and activity. Bone-derived exosomes are involved in the regulation of bone metabolism and homeostasis, influencing the activity of osteoblasts and osteoclasts [70].

### 8.1. Osteoporosis

Osteoporosis is a systemic skeletal disorder characterized by decreased bone mass and microarchitectural deterioration, leading to increased fracture risk. An earlier mouse study reported that dysregulated expression of microRNAs in EVs may contribute to osteoporosis. Exosomal miR-214-3p derived from osteoclasts inhibits osteoblastic bone formation. in vitro and in vivo studies show that miR-214-3p from osteoclasts is transferred to osteoblasts via exosomes. This transfer reduces osteoblast activity and bone formation. Moreover, inhibiting miR-214-3p in osteoclasts via osteoclast-targeted delivery promoted bone formation in ovariectomized mice, highlighting the therapeutic potential of targeting osteoclast-derived miR-214-3p in treating osteoporosis [19].

A meta-analysis revealed that compared to placebo treatment for osteoporosis, MSCs-derived EVs therapy increased bone mass, improved bone microstructure, and enhanced bone strength [71]. Both in vivo and in vitro studies confirmed that endothelial progenitor cell (EPC)-derived exosomes could promote bone repair by enhancing the recruitment and differentiation of osteoclast precursors via long non-coding RNA metastasis-associated lung adenocarcinoma transcript 1(LncRN-MALAT1) [72]. In addition, Yang et al. showed that the BMSCs-derived exosome MALAT1 enhanced osteoblast activity in osteoporotic mice by mediating the miR-34c/SATB2 axis [73]. Hu et al. discovered that EVs modified with CXCR4 exhibited bone-targeting functionality. These CXCR4-modified EVs fused with lipid nanoparticles containing Antagomir-188 and demonstrated the ability to inhibit both osteogenesis and adipogenesis, thus offering potential for the treatment of osteoporosis [74].

### 8.2. Osteoarthritis (OA)

Osteoarthritis is a degenerative joint disease characterized by cartilage degradation, synovial inflammation, and joint pain. The severity of OA and cartilage degeneration is closely associated with synovial inflammation. Both in vivo and in vitro studies have shown that exosome secretion is increased in fibroblast-like synoviocytes (FLSs) from OA patients. Notably, inflammatory FLS-derived exosomes (inf-exo) enhance M1 polarization of macrophages, inducing an OA-like phenotype in chondrocytes. Intra-articular injection of inf-exo exacerbates OA progression in murine models. Furthermore, inf-exo stimulation activates glycolysis, and inhibition of glycolysis with 2-DG reduces excessive M1 polarization. Mechanistically, HIF1A is identified as the key transcription factor, and its inhibition alleviates macrophage inflammation induced by inf-exo. In vivo administration of HIF1A inhibitors mitigates experimental OA. These findings suggest that FLS-derived exosomes contribute to OA pathogenesis by inducing macrophage dysfunction and represent a potential therapeutic target [75]. Moreover, exosomes derived from MSCs have emerged as a promising therapeutic approach for OA, a degenerative joint disease characterized by cartilage degradation and pain. Another study conducted in rabbits has reported that MSC-derived exosomes can alleviate OA symptoms by promoting cartilage repair, reducing inflammation, and improving joint function [76].

## 9. Summary and Future Direction

Tissue-derived small extracellular vesicles (sEVs/EVs) are increasingly recognized as dynamic mediators of intercellular and inter-organ communication. In the CNS, sEVs from neurons, astrocytes, microglia, and oligodendrocytes regulate synaptic function, maintain neuronal homeostasis, and modulate immune responses, yet may also propagate neurodegenerative pathology through misfolded proteins and pro-inflammatory microRNAs. Peripheral sEVs—from adipose tissue, muscle, or immune cells—can access the brain to influence neuroinflammation, synaptic activity, and cognition, highlighting a bidirectional brain–body axis.

Beyond the nervous system, EV cargos participate in metabolic, cardiovascular, oncological, and musculoskeletal disorders, underscoring their potential as cross-disease biomarkers (Table 1). From a translational perspective, sEVs are being harnessed as natural or engineered delivery vehicles, offering protection for bioactive cargos and potential tissue targeting. Preclinical studies of stem cell-derived sEVs (e.g., bone marrow, adipose, or umbilical cord MSCs) demonstrate therapeutic promise in tissue repair and immunomodulation, while engineered sEVs expand opportunities for targeted delivery [77,78,79].Representative sEV/EV-based therapeutic interventions across diseases are summarized in Table 2.

Challenges to clinical translation include EV heterogeneity, variability in isolation and characterization (MISEV2023), incomplete biodistribution and pharmacokinetic data, lack of standardized potency assays, off-target effects, and manufacturing constraints. Future efforts should focus on standardized reporting, quantitative biodistribution and trafficking, mechanistic validation of CNS–periphery signaling, and rigorous human studies benchmarking EV cargos against clinical endpoints. With these advances, tissue-derived sEVs may transition from associative biomarkers to clinically actionable diagnostics and therapeutics [80].

## Figures and Tables

**Table 1:** Representative sEV/EV-associated biomarker cargos across major diseases.

Disease/Condition	Sample Source	Key Biomarker/Cargo	Cargo Type	Clinical Utility	Refs
Alzheimer’s disease	Plasma (neuron-derived sEVs)	Aβ42/Aβ40; p-tau	protein	Diagnosis/progression monitoring	[8,9,10]
Parkinson’s disease	Plasma (erythrocyte EVs)	alpha-synuclein	protein	Risk/progression candidate	[11]
TBI	Plasma/serum	miR-21-5p; p-tau	microRNA/protein	Diagnosis/prognosis (dynamic)	[12,13]
Metabolic syndrome/T2D	Plasma; adipose EVs	miR-155/miR-34a/miR-27a; miR-126	microRNA	Risk stratification; vascular complications	[14,15]
OSA-associated atherosclerosis	Plasma EVs	endothelial activation + inflammatory cargo	mixed	Endothelial dysfunction marker	[16]
Lung cancer	Plasma EVs	let-7b-5p, let-7e-5p, miR-23a-3p, and miR-486-5p	DNA/miRNA	Early detection; therapy monitoring	[17]
HCC	Plasma EVs	microRNA/lncRNA	microRNA/lncRNA	Diagnosis; recurrence prediction	[18]
Osteoporosis	Plasma EVs	miR-214-3p	microRNA	Bone turnover monitoring	[19]

**Table 2:** Representative sEV/EV-based interventions and therapeutic cargos reported across diseases.

Condition	sEV/EV Source	Key Cargo/Pathway	Model	Therapeutic Effects	Refs
TBI	Astrocyte-derived sEVs	miR-873a-5p → NF-kappaB inhibition	mouse TBI	Reduced microglial inflammation; improved function	[40]
Repetitive mild TBI	Microglia-derived sEVs	miR-124-3p	mouse rmTBI	Reduced neuroinflammation; neuroprotection	[41,42]
TBI	MSC-derived sEVs (BMSC/UC-MSC in studies)	pro-angiogenic + anti-inflammatory miRNAs	rat TBI	Enhanced angiogenesis/neurogenesis; better outcomes	[43]
AD/PD models	Engineered/targeted sEVs	siRNA/miRNA delivery; anti-aggregation cargos	mouse/in vitro	Reduced pathological protein burden	[8,9,10]
Atherosclerosis	Endothelial/MSC sEVs	anti-inflammatory miRNAs	mouse/in vitro	Improved endothelial function; reduced plaque inflammation	[58,59,60,61]
Osteoarthritis	MSC-derived sEVs	miR-140; cartilage anabolic programs	mouse/in vitro	Cartilage protection; reduced synovitis	[75,76]

## Data Availability

No new data were created or analyzed in this study.

## Abbreviations

The following abbreviations are used in this manuscript:

EVs	extracellular vesicles
sEVs	small extracellular vesicles
BDNF	brain-derived neurotrophic factor
BBB	blood–brain barrier
AD	Alzheimer’s disease
Aβ	amyloid-β
ATEVs	adipose tissue-derived extracellular vesicles
TLR	Toll-like receptor
PD	Parkinson’s disease
AD-B-EVs	AD-derived brain EVs
BMSCs	bone marrow stromal cells
CNS	central nervous system
TBI	traumatic brain injury
MSCs	mesenchymal stem cells
NAFLD	non-alcoholic fatty liver disease
NASH	non-alcoholic steatohepatitis
T2D	type 2 diabetes
AMI	myocardial infarction
OSA	obstructive sleep apnea–hypopnea syndrome
VSMCs	vascular smooth muscle cells
HCC	hepatocellular carcinoma
TAM	tumor-associated macrophages
EOC	epithelial ovarian cancer
NSCLC	non-small cell lung cancer
TME	tumor microenvironment
OA	osteoarthritis
EPC	endothelial progenitor cells
lncRNA MALAT1	long non-coding RNA metastasis-associated lung adenocarcinoma transcript 1
FLSs	fibroblast-like synoviocytes
inf-exo	inflammatory FLS-derived exosomes
